# Small but strong: the emerging role of small nucleolar RNA in cardiovascular diseases

**DOI:** 10.3389/fcell.2023.1292925

**Published:** 2023-11-14

**Authors:** Xue Sun, Gebang Wang, Wenting Luo, Hui Gu, Wei Ma, Xiaowei Wei, Dan Liu, Shanshan Jia, Songying Cao, Yu Wang, Zhengwei Yuan

**Affiliations:** ^1^ Department of Ultrasound, Shengjing Hospital of China Medical University, Shenyang, Liaoning, China; ^2^ Key Laboratory of Health Ministry for Congenital Malformation, Shengjing Hospital of China Medical University, Shenyang, Liaoning, China; ^3^ Department of Thoracic Surgery, Liaoning Cancer Hospital and Institute, Cancer Hospital of Dalian University of Technology, Shenyang, Liaoning, China

**Keywords:** biomarker, cardiovascular disease, heart, ncRNAs, SnoRNA

## Abstract

Cardiovascular diseases (CVDs) are the leading cause of mortality and disability worldwide. Numerous studies have demonstrated that non-coding RNAs (ncRNAs) play a primary role in CVD development. Therefore, studies on the mechanisms of ncRNAs are essential for further efforts to prevent and treat CVDs. Small nucleolar RNAs (snoRNAs) are a novel species of non-conventional ncRNAs that guide post-transcriptional modifications and the subsequent maturation of small nuclear RNA and ribosomal RNA. Evidently, snoRNAs are extensively expressed in human tissues and may regulate different illnesses. Particularly, as the next-generation sequencing techniques have progressed, snoRNAs have been shown to be differentially expressed in CVDs, suggesting that they may play a role in the occurrence and progression of cardiac illnesses. However, the molecular processes and signaling pathways underlying the function of snoRNAs remain unidentified. Therefore, it is of great value to comprehensively investigate the association between snoRNAs and CVDs. The aim of this review was to collate existing literature on the biogenesis, characteristics, and potential regulatory mechanisms of snoRNAs. In particular, we present a scientific update on these snoRNAs and their relevance to CVDs in an effort to cast new light on the functions of snoRNAs in the clinical diagnosis of CVDs.

## 1 Introduction

Cardiovascular diseases (CVDs), such as ischemic heart disease, atherosclerosis, hypertension, stroke, heart failure (HF), and peripheral arterial disease, are the leading cause of morbidity and mortality worldwide, contributing greatly to a reduced quality of life (Mensah et al., 2019; Yin et al., 2022). In 2019, CVDs were the underlying cause of approximately one-third of all deaths worldwide, accounting for 9.6 million and 8.9 million deaths among men and women, respectively (Roth et al., 2020). Multiple contributing factors have been reported in the pathogenesis of CVD, including genetic factors, lifestyle, dietary risks, tobacco smoking, and obesity. Although recent economic and technical advancements in medical treatment strategies have helped reduce CVD-associated mortality rates, the risk of developing CVDs still poses a severe hazard to human society. Furthermore, while many advances have been made in the prevention, diagnosis, treatment, and prognosis of CVDs, our current understanding of the signalling pathways involved in the molecular pathophysiology thereof is limited, hampering the identification of therapeutic targets for CVDs. Thus, there is an urgent need to identify molecular pathogenesis mechanisms, potential biomarkers, and new therapeutic targets for treating CVDs.

Recently, numerous studies have demonstrated that non-coding RNAs (ncRNAs) play a primary role in CVD development (Chen et al., 2020; Smani et al., 2020; Zhu et al., 2021). Therefore, studies on the mechanisms of ncRNAs are essential to advance diagnostic, preventative, and treatment efforts for CVDs. ncRNAs can be categorised as long non-coding RNAs (lncRNAs) and small non-coding RNAs (sncRNAs), the latter of which comprise microRNAs (miRNAs), transfer RNAs (tRNAs), small nuclear RNA (snRNAs), piwi-interacting RNAs (piRNAs), and small nucleolar RNAs (snoRNAs) (Bergeron et al., 2020; Li X et al., 2021). In particular, snoRNAs are short (60–300 nucleotides) essential sncRNAs that primarily reside in the nucleolus of eukaryotic cells (Li M et al., 2021). Their localisation indicates a direct link to their conventional function: which is to guide post-transcriptional modifications and the subsequent maturation of rRNAs and snRNAs (Bergeron et al., 2020). snoRNAs can be detected stably in plasma and serum, and blood snoRNA levels indicate different disease statuses and relate to clinicopathological features, highlighting their suitability as disease biomarkers (Liao et al., 2010; Okugawa et al., 2017). Emerging evidence has demonstrated that snoRNAs have the potential to serve as biomarkers for a variety of diseases. For example, SNORA31 variations impair cortical neuron-intrinsic immunity to herpes simplex virus-1 (HSV-1) and underlie herpes simplex encephalitis (HSE), thereby providing a new genetic aetiology and immunological mechanism of HSE (Lafaille et al., 2019). Additionally, SNORD46 overexpression has been demonstrated to promote cancer cell growth, migration, and invasion (Gong et al., 2017). A plasma SNORD33 signature serves as a predictor of platinum-based chemotherapy sensitivity in patients with metastatic triple-negative breast cancer, which may have immediate translational relevance for precision platinum-based chemotherapy (Wang et al., 2022).

snoRNAs are extensively expressed in various mammalian tissues, including the brain, heart, reproductive organs, lungs, skeletal muscle, and liver (Watson et al., 2019; Fafard-Couture et al., 2021; Wei et al., 2021; Wu et al., 2022). Increasing evidence suggests that snoRNAs contribute to the development of CVDs such as congenital heart disease (CHD) (O’Brien et al., 2012), coronary heart disease (Mick et al., 2017), myocardial infarction (MI) (Hakansson et al., 2019), and HF(Hakansson et al., 2019). These reports have gained gradual acclaim by contributing to our understanding of the emerging role of snoRNAs in CVDs.

Considering this, this systematic review comprehensively collated available PubMed literature on the classification, characteristics, biogenesis, and potential regulatory mechanisms of snoRNAs in CVDs using the search terms “small nucleolar RNA,” “snoRNA,” “cardiovascular disease,” “CVD,” “heart,” “cardiac,” “RNA-seq,” and “transcriptomics” (an exhaustive list of all keywords searched can be found in the supplementary material). In particular, we present a scientific update on snoRNAs and their relevance to CVDs in an effort to cast new light on the functions of snoRNAs in the clinical diagnosis of CVDs.

## 2 Classification and characteristics of snoRNAs

snoRNAs can be categorized into two main structural classes based on their structure, conserved sequence, and rRNA modification functions (Huang W et al., 2022), C/D Box snoRNA (Massenet et al., 2017; Yu et al., 2018; Baldini et al., 2021) and H/ACA Box snoRNA (Stepanov et al., 2015; Caton et al., 2018; Trucks et al., 2021). C/D Box snoRNA and H/ACA Box snoRNA families predominate and have been exhaustively researched, whereas limited literature exist on small Cajal body (CB)-specific RNAs (scaRNAs) (Deryusheva and Gall, 2013; Beneventi et al., 2021) and orphan snoRNAs (Kocher et al., 2021; Xu et al., 2021). The structural characteristics of each snoRNA type are delineated below.

### 2.1 C/D box snoRNA

**FIGURE 1:**
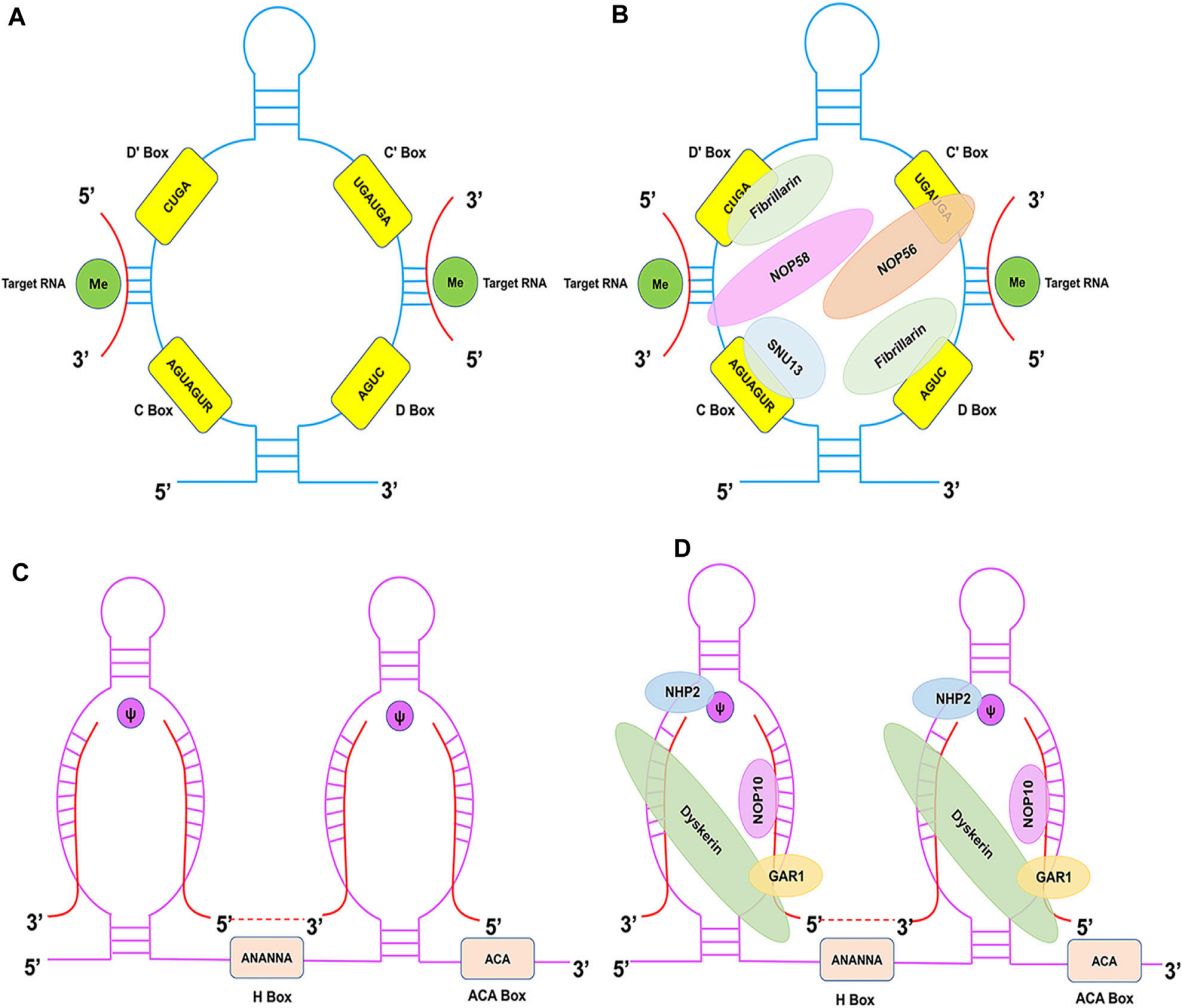
Structural features of C/D Box and H/ACA Box snoRNAs and associated proteins. **(A)** The C/D Box snoRNA consists of a 5′ C Box (RUGAUGA), a 3′ D Box (CUGA), and the C′ and D′ Boxes located internally. **(B)** The C/D Box snoRNP complex consists of NOP56 (orange), NOP58 (pink), SNU13 (blue), and Fibrillarin (green) proteins. **(C)** The H/ACA Box snoRNA consists of ACA Box (ACA) and H Box (ANANNA) located in the 3′-end and the hinge region, respectively. **(D)** The H/ACA Box snoRNP complex consists of NHP2 (blue), NOP10 (pink), GAR1 (yellow), and Dyskerin (green) proteins.

C/D Box snoRNAs are typically 60–90 nucleotides in average length and are characterised by the presence of two conserved sequence motifs near their 5′ and 3′ termini: C Box (RUGAUGA) and D Box (CUGA), respectively (Figure 1A) (Baldini et al., 2021). The C and D Boxes comprise a kink-turn motif (k-turn) with noncanonical G-A base pairing and results in a severe bend in the axis of the double-stranded RNA molecule. The k-turn, a scaffold for the assembly of C/D Box ribonucleoproteins (snoRNPs), such as NOP56, NOP58, Fibrillarin, and SNU13 (15.5 kDa), is crucial for biogenesis and correct localisation (Figure 1B) (Massenet et al., 2017). This characteristic structure has biological significance in the processing and formation of snoRNAs, maintenance of stability, methylation modification, and localisation in the nucleolus. A second pair of C′ and D′ Boxes are localised closer to the middle of the C/D Box molecule, yet they often display lower sequence conservation than the C and D Boxes (Yu et al., 2018). The guide region (10–20 nucleotides) is found immediately upstream of the D Box and/or D′ Box, and it ensures modification specificity by base pairing with the target RNA.

### 2.2 H/ACA box snoRNA

H/ACA Box snoRNAs (120–140 nucleotides) are characterised by the hallmark ‘hairpin-hinge-hairpin-tail’ secondary structure, which consists of two hairpin domains connected by single-stranded (hinge) and 3′-terminus (tail) regions (Stepanov et al., 2015). The H Box (ANANNA) and ACA Box are located close to the hairpin in the hinge and tail regions, respectively (Figure 1C) (Trucks et al., 2021). The H/ACA Box snoRNA antisense region is composed of two short (9–16 nucleotides) sequences located in the internal loops of the 5′ and/or 3′ hairpins. Typically, a bulge on the hairpins forms the pseudouridylation pocket, and these regions harbour complementarity with target RNAs. The H/ACA Box forms a snoRNP with Dyskerin (pseudouridine synthase), NHP2, NOP10, and GAR1 (Figure 1D) (Caton et al., 2018). Dyskerin directly promotes pseudouridylation, whereas NOP10 and NHP2 contribute to the stability and function of H/ACA Box snoRNAs. Moreover, GAR1 enhances the catalytic activity of Dyskerin and facilitates the release of the target RNA. This characteristic structure is essential for snoRNA biogenesis, stability, and localisation.

### 2.3 scaRNA

Another subfamily of snoRNAs, namely, scaRNAs, is localised in the subnuclear CBs and possesses similar structural features to that of either C/D Box snoRNA, H/ACA Box snoRNA, or both. scaRNAs harbour additional nucleotide motifs, namely, GU-repeat bound and CAB boxes (UGAG), that function as CB localisation signals. Furthermore, scaRNAs not only have many similar functions to that of other snoRNAs, but also guide 2′-O-methylation and pseudouridylation of U1, U2, U4, and U5 snRNAs (Deryusheva and Gall, 2013). Moreover, scaRNA modification can occur in the nucleolus rather than the CB. Hence, they examine the possibility that scaRNA processing is not limited to the CB.

### 2.4 Orphan snoRNA

It has been shown that numerous C/D and H/ACA Box snoRNAs, referred to as orphan snoRNAs, lack modification targets among rRNAs or snRNAs and have unknown functions (Kocher et al., 2021; Xu et al., 2021). For example, Xu et al. found that SNORD126 interacts with hnRNPK protein to regulate *FGFR2* transcription, and activate the PI3K-AKT pathway (Xu et al., 2021). They confirm that SNORD126 plays a significant role in the development of hepatocellular carcinoma and propose that inhibiting SNORD126 expression could be an effective therapeutic strategy for treating hepatocellular carcinoma. This finding suggests that orphan snoRNAs have important physiological significance.

## 3 Biogenesis and function of snoRNAs

**FIGURE 2:**
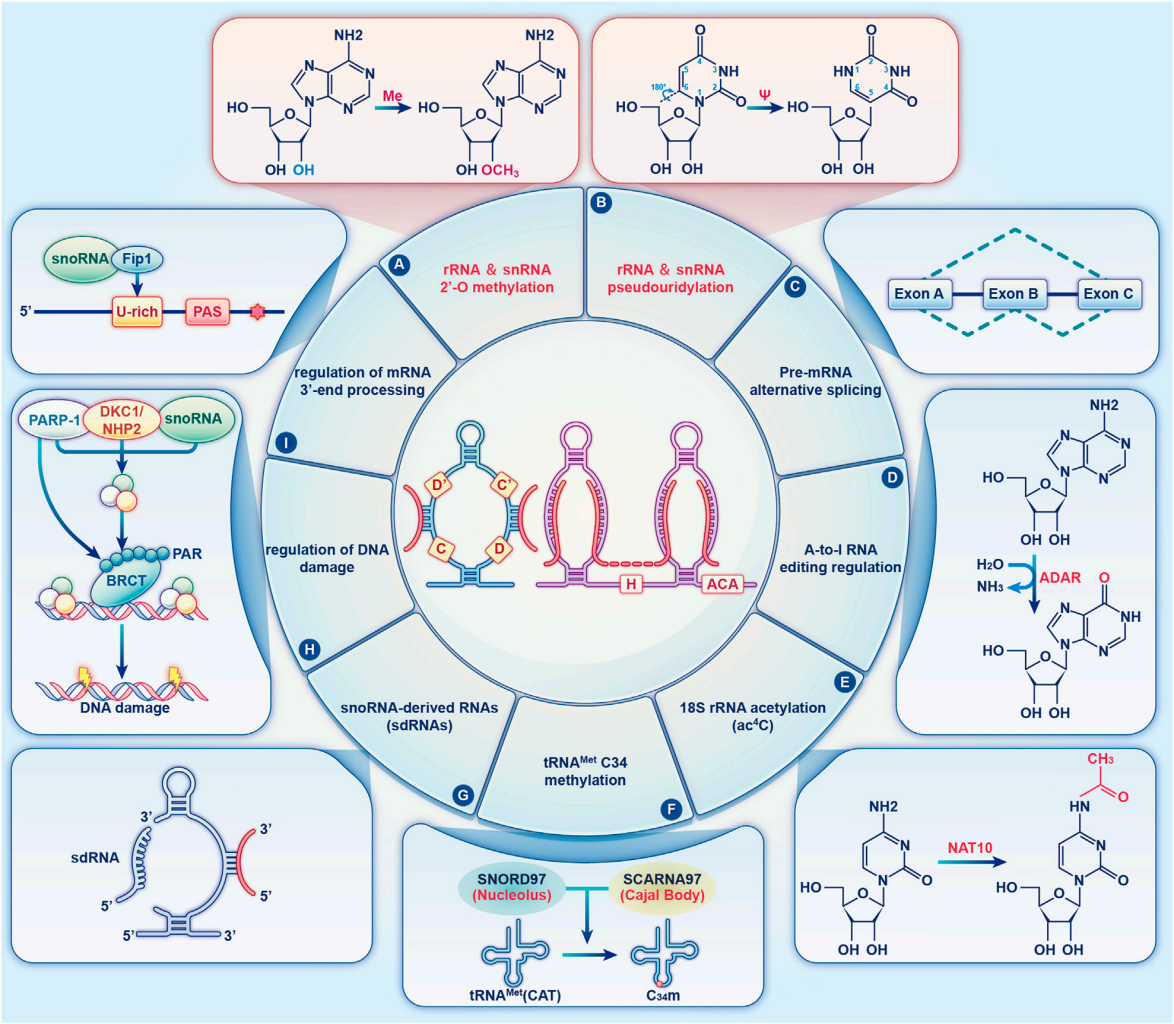
The diverse functions of snoRNAs. An overview of the diverse functions of snoRNAs that are described in the text. The two canonical functions of snoRNAs are realised by guiding the 2′-O-methylation **(A)** and pseudouridylation **(B)** of rRNA and snRNA (red). **(C)** Orphan snoRNAs play key roles in alternative splicing. **(D)** snoRNAs may be involved in A-to-I RNA editing. **(E)** snoRNAs play a role in 18S rRNA acetylation. **(F)** SNORD97 snoRNA and its CB-specific functional homologue, SCARNA97, can direct 2′-O-methylation of the wobble cytidine C34 of human elongator tRNA^Met^ (CAT) in a cooperative fashion. **(G)** snoRNAs can be processed to generate snoRNA-derived RNAs (sdRNAs), which have been shown to perform a regulatory function similar to that of miRNAs or piRNAs. **(H)** snoRNAs are enriched in chromatin, which affects DNA damage and genomic stability. **(I)** Specific snoRNAs physically associated with the mRNA 3′-end processing complex, affecting the targeting of polyadenylation sites.

snoRNAs are either directly transcribed from snoRNA genes via independent promoters, or they are encoded by intronic snoRNA genes without independent promoters (Bratkovic et al., 2020). snoRNAs serve a crucial function in the machinery of protein synthesis. The canonical functions of C/D and H/ACA Box snoRNAs are realised by respectively guiding the 2′-O-methylation and pseudouridylation of rRNA and snRNAs in a base-pair-dependent manner (Cao et al., 2018). In the past 20 years, novel snoRNAs have been discovered, and unexpected functions have been identified for previously annotated snoRNAs, which has led to the discovery of their diverse roles. Several studies suggest that snoRNAs regulate cell physiology by additional non-canonical modifications, including regulating alternative splicing (Nilsen and Graveley, 2010), adenosine-to-inosine (A-to-I) editing (Heraud-Farlow and Walkley, 2020), tRNA^Met^ C34 methylation (Vitali and Kiss, 2019), DNA damage (Han et al., 2022), and mRNA 3′-processing (Kumar et al., 2019), guiding N4-acetylcytidine (ac4C) modifications (Jin et al., 2020), and performing snoRNA-derived RNA (sdRNA) functions (Wajahat et al., 2021) (Figure 2). Below, we review some of the key functions of snoRNAs by focussing on their mechanism of action.

### 3.1 Canonical functions of snoRNA

#### 3.1.1 2′-O-methylation

When active in its dimeric form, C/D Box snoRNA guides 2′-O-methylation of target rRNAs (Figure 2A) (Cao et al., 2018). Specifically, snoRNAs form a double helix by binding a complementary sequence in rRNA, and the nucleotide opposite the fifth nucleotide is always placed upstream of the snoRNA D or D′ Boxes. Fibrillarin is an important component of snoRNPs that transfers the methyl group from S-adenosyl methionine (SAM) to the 2′-hydroxyl group in the target RNA (Dennis et al., 2015). 2′-O-methylation optimises the structure of rRNA, thereby increasing its stability (Monaco et al., 2018).

#### 3.1.2 Pseudouridylation

Pseudouridylation is a highly prevalent RNA post-transcriptional modification and is guided by H/ACA Box snoRNAs (Figure 2B) (Liang and Li, 2011). This reaction, which is catalysed by the pseudouridylase Dyskerin (Garus and Autexier, 2021), maintains RNA stability and regulates ribosome synthesis (Borchardt et al., 2020). By binding to the target RNA, H/ACA Box snoRNAs convert the target uridine into pseudouridine, thereby increasing the target specificity. Additionally, pseudouridine was found to stabilise its adjacent nucleotides, which may influence the fidelity of the ribosome (King et al., 2003).

### 3.2 Non-canonical functions of snoRNA

#### 3.2.1 Alternative splicing

Alternative splicing (AS) is primarily regulated by RNA-binding proteins that bind pre-mRNAs near different splice sites to produce variably spliced mRNAs (Figure 2C) (Nilsen and Graveley, 2010). AS enriches the transcriptional output in eukaryotes and is important for gene expression (Leung et al., 2021). Different combinations of exons are created through AS to generate a variety of mRNAs, thereby increasing the variety of proteins expressed either in different tissues or in the same tissue at different intervals. Therefore, AS allows the production of mRNAs with different structures and functions, resulting in different encoded proteins (Pan et al., 2008). Chen et al. demonstrated that SNORA70E regulates the AS of *PARPBP* to promote the development of ovarian cancer (Chen et al., 2022). Falaleeva et al. found SNORD27 regulates the AS of the transcription factor *E2F7* pre-mRNA through direct RNA-RNA interaction (Falaleeva et al., 2016). These findings suggest that snoRNAs likely affect the regulation of AS.

#### 3.2.2 A-to-I RNA editing regulation

A-to-I RNA editing, a unique type of post-transcriptional RNA modification, is a chemical base change that results the alteration of the primary nucleotide sequences (Heraud-Farlow and Walkley, 2020). In mammals, there are two common forms of RNA editing: A-to-I and cytidine to uridine (C-to-U). The former is catalysed by a family of enzymes called adenosine deaminases acting on RNA (ADARs) (Figure 2D) (Yang et al., 2021; Niescierowicz et al., 2022). Recent evidence indicates the capacity of A-to-I editing to modulate snoRNA precursors. Huang et al. discovered LNC-SNO49AB, a new and highly expressed snoRNA-related lncRNA that interacts with ADAR1 to enhance dimerization and increase RNA A-to-I editing rates, thereby regulating hematopoietic malignancy (Huang Z H et al., 2022).

#### 3.2.3 18S rRNA acetylation

As a highly conserved RNA modification catalysed by NAT10 (human) or Kre33 (yeast) (Jin et al., 2020), ac4C can be found in mRNAs, rRNAs, and tRNAs (Figure 2E). The 18S rRNA of eukaryotes contains two acetylated cytidines located in helices 34 and 45, which are crucial for translation fidelity and are part of the ribosome decoding site, respectively (Sharma et al., 2015). Sharma et al. showed that the acetylation of two N4 cytidine residues in 18S rRNA is mediated by two orphan C/D Box snoRNAs (snR4 and snR45) that direct the RNA cytidine acetyltransferase Kre33 to the target site (Sharma et al., 2017). Moreover, direct interactions between snR45 and snR4 were detected in yeast 18S rRNA using CLASH(Dudnakova et al., 2018), and the interaction of SNORD13 (the vertebrate homologue of snR45) with 18S rRNA was confirmed using PARIS (Lu et al., 2016). Bortolin-Cavaillé et al. found an atypical *Drosophila melanogaster* SNORD13 homologue in which a single antisense strand is sufficient for ac4C deposition, while the second may enhance the ac4C efficiency (Bortolin-Cavaille et al., 2022). Accordingly, a novel approach was revealed for reconstituting RNA-guided cytidine acetylation in human cells, thereby providing new insights into understanding and using RNA-guided RNA modification catalysts in biotechnology and disease.

#### 3.2.4 tRNA^Met^ C34 methylation

tRNAs undergo a variety of post-transcriptional modifications with rich diversity, pointing their role in stabilising tRNA-mRNA interactions during decoding (Jackman and Alfonzo, 2013). Recently, Vitali and Kiss demonstrated that SNORD97 and its Cajal body functional homolog SCARNA97 (previously known as SNORD133), showing strong sequence complementarity among the two snoRNA guide regions and the tRNA^Met^ (CAT) around the wobble cytidine C34, which was directed to be 2′-O methylated (Figure 2F) (Vitali and Kiss, 2019). Moreover, 2′-O-methylation of C34 inhibited tRNA^Met^ (CAT) into tRNA fragments (tRFs) via the stress-responsive endoribonuclease angiogenin. This implies a regulatory connection between RNA-directed tRNA modification and angiogenin-mediated tRF processing (Nostramo and Hopper, 2019). It might be a new insight to discovery of tRNA instead of rRNA or snRNA as snoRNA and scaRNA targets; however, likely only the tip of the iceberg and further in-depth investigation is required to comprehensively elucidate this connection.

#### 3.2.5 snoRNA-derived RNAs

Using RNA-seq analyses, numerous groups have reported the fragmented profiles of several snoRNAs. These fragments can be processed to generate small lineage-specific conserved and functional RNAs, namely, sdRNAs (Figure 2G) (Wajahat et al., 2021; Coley et al., 2022b). These sdRNAs have miRNAs or piRNAs characteristics, which widen the functional roles of snoRNAs in a biological context. The first report of this identified that snoRNA ACA45 is processed by Dicer into small RNAs (Ender et al., 2008). These small RNAs have miRNA-like functions through which they target CDC2L6. This clearly demonstrated the cytoplasmic role of snoRNA derivatives. Additionally, in TCGA prostate cancer (PCa) patient samples, Coley et al. discovered 38 specifically excised, differentially expressed sdRNAs, which sdRNA-A24 and sdRNA-D19b were the most differentially expressed among the different fragments (Coley et al., 2022a). Therefore, they believed that these sdRNAs could potentially serve as new Pca biomarkers and therapeutic targets. Moreover, He et al. demonstrated that a Growth Arrest Specific 5 (*GAS5*)-derived piRNA induced H3K4 methylation/H3K27 demethylation to upregulate the transcription of tumour necrosis factor-related apoptosis-inducing ligand (TRAIL) (He et al., 2015). However, the precise mechanisms and functionality of sdRNAs remain to be elucidated.

#### 3.2.6 Regulation of DNA damage

Chromatin-associated RNAs (caRNAs) are a recently identified class of RNA molecules that are linked to chromatin transcription. Moreover, caRNAs are involved in a variety of biological processes, including the regulation of genes, chromatin structure, cell division, and cell specification (Diermeier et al., 2014). Recent evidence has shown that a fraction of snoRNAs is enriched in caRNAs from human and *Drosophila* cells, forming an RNP complex that may play a role in eukaryotic chromatin biology. For example, Schubert et al. indicated that snoRNAs are associated with *Drosophila* chromatin-binding protein (Df31), which plays a role in maintaining open chromatin structure (Schubert et al., 2012). Another study found that snoRNA bind poly ADP-ribose polymerase 1 (PARP1) to stimulate PARP-1 ADPRylates DDX21 in cells to promote cell proliferation (Kim et al., 2019). Han et al. found that SNORA73, which is enriched in chromatin, suppressed PARP1 auto-PARylation by forming a non-canonical snoRNP with PARP1 and the canonical partners DKC1/NHP2 to affect DNA damage and genome stability (Figure 2H) (Han et al., 2022). Mechanistically, SNORA73’s 5′ non-canonical structure is essential for its function and PARP1 binding. Their findings emphasised the potential chromatin-associated functions of snoRNAs and provided novel insights into the functional diversity of snoRNAs in cells.

#### 3.2.7 Regulation of mRNA 3′-end processing

mRNA 3′-end processing occurs cotranscriptionally and is an essential step in gene expression (Kumar et al., 2019). Huang et al. found a subset of snoRNAs physically associated with the mammalian mRNA 3′-processing complex. SNORD50A is U/A-rich and specifically interacts with FIP1, thereby preventing FIP1 from interacting with poly(A) site (PAS) sequences *in vitro* and *in vivo* (Figure 2I) (Huang et al., 2017). Of note, they provided the first experimental evidence that snoRNAs may play a regulatory role; however, they are not essential in regulating mRNA 3′-end processing. Nevertheless, their findings provide novel insights into the regulatory mechanism of mRNA 3′-end processing and expand our understanding of the non-canonical functions of snoRNAs.

## 4 Detection, analysis and databases of snoRNAs

snoRNAs, as a class of ncRNAs, often show heterogeneity regarding their structure, interacting proteins, RNA modification types and targeting locations. Therefore, sophisticated and collaborative profiling approachs to analyse their role are required. Numerous methods, including qPCR(Galiveti et al., 2010), RNA-seq (Wu et al., 2016), Northern blotting (Kishore et al., 2010) and RNA protection assay (RPA), are commonly utilized to analyze the expression of snoRNA at present. The snoRNA-protein interactions are promising including UV crosslinking and immunoprecipitation (CLIP) (Cao et al., 2019), RNA immunoprecipitation (RIP), RNA antisense purification (RAP) (Cao et al., 2019), RNA pull-down (Li et al., 2020), and Cross-linking and analysis of cDNA (CRAC) (Cao et al., 2019) techniques. RNA hybrid and individual nucleotide resolution CLIP (hiCLIP) (Cao et al., 2019), and crosslinking, ligation, and sequencing of hybrids (CLASH) (Cao et al., 2019) methods are used to analyze the RNA-RNA interactions.

For future studies on snoRNAs, several databases can be used such as snoRNABase, snoRNA Atlas, snoPY, and snoDB (Bergeron et al., 2023). Researchers can use to analyze snoRNA sequences, features, host gene, chromosomal location, average abundance, snoRNA-RNA targets, and snoRNA orthology relationships to fill gaps in snoRNA research.

## 5 Role of snoRNAs in CVD

**FIGURE 3:**
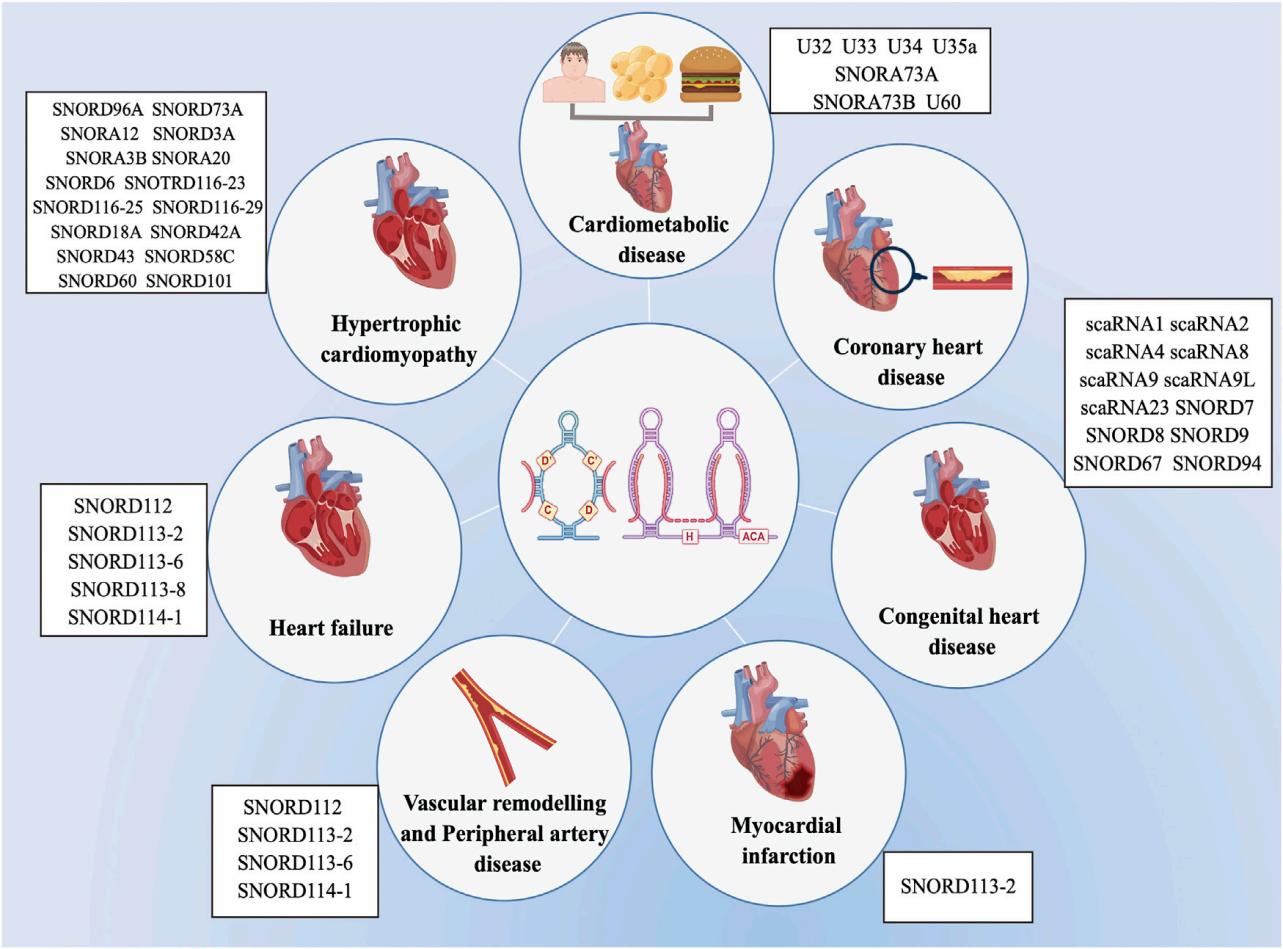
List of snoRNAs that differentially expressed in cardiovascular diseases. The dysregulation of snoRNAs contributes to the progression of various cardiovascular diseases, including cardiometabolic disease, coronary heart disease, congenital heart disease, myocardial infarction, vascular remodelling and peripheral artery disease, heart failure and hypertrophic cardiomyopathy.

Previous researches on the biological roles of regulating snoRNAs have primarily focused on the development of various cancers (Mourksi et al., 2020; Ding et al., 2021; Zhu et al., 2022). Nevertheless, snoRNAs have many characteristics, especially tissue or cell specificity. In recent studies, snoRNAs have been found in the cardiovascular tissues and are differentially expressed in patients with CVD (O’Brien et al., 2012; Mick et al., 2017; Nossent et al., 2019). These findings have revealed the essential role that snoRNAs play in cardiac development, pathophysiology, and lipid metabolism, as well as reveal their potential as both biomarkers of disease and novel therapeutic targets (Table 1) (Michel et al., 2011; Rajan et al., 2017; Das et al., 2020; Killick et al., 2022), however, the associated regulatory mechanisms have not been comprehensively investigated. Limited research exist on the functions of snoRNAs in CVDs, and to date, no systematic review has collated the broad range of topics on snoRNAs in CVDs. Therefore, we provide an extensive overview of the latest research on snoRNAs identified in a variety of CVDs (Figure 3).

**TABLE 1:** The function and mechanisms of snoRNAs in cardiovascular diseases.

Disease	Regulation	snoRNA name	Class	Genomic location	Host gene	Target gene	Mechanism/Pathway	Function	References
CMD	Dynamically regulate	U32(SNORD32A)	C/D	19q13.33	RPL13A	18S rRNA	Regulated by NADPH oxidase	Unknown	Michel et al., 2011; Holley et al., 2015
	Dynamically regulate	U33(SNORD33)	C/D	19q13.3	RPL13A	18S rRNA	Regulated by NADPH oxidase	Unknown	Michel et al., 2011; Holley et al., 2015
	Dynamically regulate	U34(SNORD34)	C/D	19q13.33	RPL13A	28S rRNA	Regulated by NADPH oxidase	Unknown	Michel et al., 2011; Holley et al., 2015
	Dynamically regulate	U35a (SNORD35A)	C/D	19q13.33	RPL13A	28S rRNA	Regulated by NADPH oxidase	Unknown	Michel et al., 2011; Holley et al., 2015
	Dynamically regulate	SNORA73A (U17a)	H/ACA	1p35.3	SNHG3(U17HG)	unknown	Regulate HUMMR, regulate cell metabolism through mTOR pathway	Unknown	Jinn et al., 2015; Sletten et al., 2021
	Dynamically regulate	SNORA73B(U17b)	H/ACA	1p35.3	SNHG3(U17HG)	unknown	Regulate HUMMR, regulate cell metabolism through mTOR pathway	Unknown	Jinn et al., 2015; Sletten et al., 2021
	Dynamically regulate	U60(SNORD60)	C/D	16p13.3	Cluster of ESTs	28S rRNA	Affecting internalization of plasma membrane cholesterol	Unknown	(Brandis et al., 2013)
CHD	Downregulated	scaRNA9	C/D	Chr11	KIAA1731	U2	Unknown	AS	(O'Brien et al., 2012)
	Downregulated	scaRNA2	C/D	Chr1	—	U2	Unknown	AS	(O'Brien et al., 2012)
	Downregulated	scaRNA8	H/ACA	Chr9	FAM29A	U2	Unknown	AS	(O'Brien et al., 2012)
	Downregulated	scaRNA4	H/ACA	Chr1	KIAA0907	U2	Unknown	AS	(O'Brien et al., 2012)
	Downregulated	scaRNA1 (ACA35)	H/ACA	Chr1/1p35.3	PPP1R8	U2	Alter pseudouridylation at U^89^ in U2	AS	O'Brien et al., 2012; Patil et al., 2015; Nagasawa et al., 2018; Nagasawa et al., 2020
	Downregulated	SNORD7	C/D	Chr17/17q12	Cluster of ESTs	U6	Unknown	AS	(O'Brien et al., 2012)
	Downregulated	SNORD8	C/D	Chr14	CHD8	U6	Unknown	AS	(O'Brien et al., 2012)
	Downregulated	SNORD9	C/D	Chr14	CHD8	U6	Unknown	AS	(O'Brien et al., 2012)
	Downregulated	SNORD67	C/D	Chr11	CKAP5	U6	Unknown	AS	(O'Brien et al., 2012)
	Downregulated	SNORD94	C/D	Chr2	PTCD3	U6	Alter methylation at C^62^ in U6	AS	O'Brien et al., 2012; Patil et al., 2015; Nagasawa et al., 2018; Ogren et al., 2019
	Downregulated	scaRNA23	H/ACA	ChrX	POLA1	U6	Unknown	AS	(O'Brien et al., 2012)
	Downregulated	scaRNA9L	C/D	ChrX	EIF1AX	U2	Unknown	AS	(O'Brien et al., 2012)
HF	Upregulated	SNORD112	C/D	14q32	MEG8	Unknown	Unknown	Unknown	(Hakansson et al., 2019)
	Upregulated	SNORD113-2	C/D	14q32	MEG8	Unknown	Unknown	Unknown	(Hakansson et al., 2019)
	Upregulated	SNORD113-6	C/D	14q32	MEG8	Unknown	Unknown	Unknown	(Hakansson et al., 2019)
	Upregulated	SNORD113-8	C/D	14q32	MEG8	Unknown	Unknown	Unknown	(Hakansson et al., 2019)
	Upregulated	SNORD114-1	C/D	14q32	MEG8	Unknown	Unknown	Unknown	(Hakansson et al., 2019)
MI	Upregulated	SNORD113-2	C/D	14q32	MEG8	Unknown	Unknown	Unknown	(Hakansson et al., 2019)
PAD	Upregulated	SNORD112	C/D	14q32	MEG8	Unknown	Unknown	Unknown	(Nossent et al., 2019)
	Upregulated	SNORD113-2	C/D	14q32	MEG8	Unknown	Inverse correlations with leukocyte counts, active smoking and platelet activation	Unknown	(Nossent et al., 2019)
	Upregulated	SNORD 113-6	C/D	14q32	MEG8	Unknown	Unknown	Unknown	(Nossent et al., 2019)
	Upregulated	SNORD 114-1	C/D	14q32	MEG8	Unknown	Inverse correlations with leukocyte counts, active smoking and platelet activation	Unknown	Håkansson et al., 2018; Nossent et al., 2019
HCM	Upregulated	SNORD96A	C/D	5q35.3	GNB2L1	5.8S rRNA	Unknown	PTMs and AS	(James et al., 2021)
	Upregulated	SNORD73A	C/D	4q31.3	RPS3A	28S rRNA	Unknown	PTMs and AS	(James et al., 2021)
	Upregulated	SNORA12	H/ACA	10q24.31	CWF19L1	Unknown	Unknown	Unknown	(James et al., 2021)
	Upregulated	SNORD3A	C/D	17p11.2	—	Unknown	Unknown	Unknown	(James et al., 2021)

AS, alternative splicing; CHD, congenital heart disease; CMD, cardiometabolic disease; HCM, hypertrophic cardiomyopathy; HF, heart failure; MI, myocardial infarction; PAD, peripheral artery disease; PTM, post translational modifications.

### 5.1 Role of snoRNAs in cardiometabolic disease

Cardiometabolic diseases (CMDs) are a group of metabolic abnormalities that increases the risk of CVDs and type 2 diabetes mellitus (T2DM), in addition to pathological changes such as hypertension, hyperglycemia, lipid metabolism abnormalities, and environmental risk factors like poor diet, sedentary lifestyle, and smoking, which are the main causes of frailty, disability, and decreased quality of life in the older (Ash-Bernal and Peterson, 2006; Killick et al., 2022; Kim et al., 2022). Several studies have investigated the function of snoRNAs in CMDs, including diabetes mellitus, lipid metabolism abnormalities, and doxorubicin cardiotoxicity (Michel et al., 2011; Amri and Scheideler, 2017; Schaffer, 2020). The first of these studies found that the loss of three snoRNAs (U32a, U33 and U35a) from the ribosomal protein L13a (*RpL13a*) locus conferred *in vitro* resistance to lipotoxic and oxidative stress and prevented the *in vivo* propagation of oxidative stress (Michel et al., 2011). These snoRNAs are not only important oxidative stress regulators, but also perform a physiological function in increasing oxidative stress in response to lipid toxicity and systemic inflammation. These findings were the first to implicate snoRNAs as regulators of metabolic stress responses in mammalian cells. Moreover, they demonstrated that *RpL13a* snoRNAs accumulate in the cytoplasm during lipotoxic or oxidative stress (Holley et al., 2015). Doxorubicin-induced oxidative stress is dynamically regulated by NADPH oxidase, which causes the rapid cytoplasmic accumulation of *RpL13a* snoRNAs during lipotoxic or oxidative stress. These findings imply that snoRNAs may coordinate the response to environmental stress through molecular interactions outside the nucleus. Lee et al. showed that the loss of *RpL13a* snoRNAs affected mitochondrial metabolism and reduced reactive oxygen species tone, which increased insulin secretion from pancreatic islets and improved systemic glucose tolerance (Lee et al., 2016). After inflammation, *RpL13a* snoRNAs have also been detected in extracellular vesicles (EVs), suggesting that they may be picked up by other cells to guide 2′-O-methylation (Rimer et al., 2018). Similarly, both SNORA73 (U17) and SONRD60 (U60) directly regulate lipid metabolism (Brandis et al., 2013; Jinn et al., 2015; Sletten et al., 2021). Furthermore, SNORA73 regulates cholesterol homeostasis of cells by inhibiting the expression of hypoxia-upregulated mitochondrial movement regulator (HUMMR). This SNORA73-HUMMR pathway may play a physiological role in gonadal tissue maturation (Jinn et al., 2015). Sletten et al. found that *in vivo* knockdown of SNORA73 prevents lipid-induced hepatic steatosis, oxidative stress, and inflammation (Sletten et al., 2021). These findings suggest that snoRNAs could serve as therapeutic targets for metabolic diseases. In addition, SNORD60 contributes to cholesterol homeostasis (Brandis et al., 2013). In summary, these snoRNAs may play a critical role in CMDs by affecting cholesterol metabolism, reactive oxygen species, oxidative stress, inflammation, and diabetes mellitus.

### 5.2 Role of snoRNAs in CHD

CHD is a predominant birth defect that affects approximately 1.9%–7.5% of live births (Williams et al., 2019; Kruszka and Beaton, 2020; Kim, 2021; Samad and Wu, 2021). CHD contains a wide scope of heart malformations, ranging from a single abnormality (atrial septal defect or ventricular septal defect) to complicated multiple defects (hypoplastic left heart syndrome or tetralogy of Fallot) (Morton et al., 2022). Tetralogy of Fallot (TOF) is the most common cyanotic CHD (Zeng et al., 2016; Morgenthau and Frishman, 2018). Recent studies comparing the snoRNAs in the right ventricular myocardium of infants with TOF to those of normal foetal and developing infant hearts suggested that the expression of 135 snoRNAs (including 12 scaRNAs) were statistically distinct in the TOF myocardium (O’Brien et al., 2012). Most of these snoRNAs (*n* = 126) showed decreased expression levels in the TOF myocardium. The expression of 115 snoRNAs decreased similarly in the foetal myocardium and the control tissue. The majority of the downregulated snoRNAs targeted four ncRNAs: rRNAs 28S and 18S, and two snRNAs, namely, U2 and U6. However, the 12 scaRNAs that were substantially downregulated in the TOF myocardium targeted only U2 and U6. Furthermore, U2 and U6 exhibited lower expression levels in TOF and foetal tissues compared to those of the control. Notably, approximately 7.5% of all genes bearing U2-type intron/exon recognition sequences displayed variation in their splice forms, and nearly 51% of genes associated with heart development were alternatively spliced in TOF compared with their controls. Taken together, these results indicate a connection between the downregulated expression of scaRNAs targeting the snRNAs U2 and U6, alternative splicing, and developmental defects in infants with TOF. These novel insights into the altered expression of snoRNAs in infants with TOF suggest that snoRNAs may affect the expression of important genes, transcript splicing, and translation during development, thereby possibly leading to the development of cardiac defects. Patil et al. and Nagasawa et al. inhibited scaRNA expression in cell cultures, resulting in abnormal splicing of several genes that regulate heart development (e.g., *gata4*, *mbnl1*, and Wnt-pathway genes) (Patil et al., 2015; Nagasawa et al., 2018). This may be ascribed to the impairment of snRNA modifications as a result of the inhibited scaRNA expression. Thereafter, they targeted the knockdown of two scaRNAs (SNORD94 and scaRNA1) in zebrafish that resulted in abnormal heart development and the alteration of splice isoforms of cardiac regulatory genes. These observations strongly suggest that snoRNAs and snRNAs are necessary for normal cardiac development. Furthermore, Ogren et al. reported that methylation in the target region of SNORD94 on U6 is decreased in right ventricular myocardium tissue of infants with TOF compared with that of the control (Ogren et al., 2019). Additionally, cell culture experiments revealed that, by changing the levels of SNORD94, a corresponding change in methylation at C^62^ within the snRNA U6 is produced. Taken together, these findings suggest a direct relationship between scaRNA expression and target nucleotide methylation. Moreover, scaRNA influences alternative splicing of mRNA; however, further investigation is needed to comprehensively elucidate this mechanism and its underlying pathway.

Nagasawa et al. further investigated the relationship between scaRNA1 and pseudouridylation levels in snRNA U2 (Nagasawa et al., 2020). Their results showed that pseudouridylation levels were significantly decreased in the right ventricular myocardium tissue of infants with TOF compared with that of the control. Moreover knockdown of scaRNA1 levels in normal primary cardiomycytes resulted in a significant decrease of pseudouridylation. This indicates that scaRNA1 levels determine the pseudouridylation levels of uridine 89 (U^89^) in snRNA U2 and affect alternative mRNA splicing and embryonic development. Based on these heart development studies, we conclude that snoRNAs are important in cardiac development and CHDs.

### 5.3 Role of snoRNAs in coronary heart disease

Coronary heart disease is a fatal CVD that is difficult to clinically diagnose early (Yin et al., 2021). Coronary heart disease is characterized by stenosis or occlusion of blood vessels due to coronary atherosclerosis, which impedes blood flow to the myocardium and results in myocardial ischemia, hypoxia, and necrosis (Boudoulas et al., 2016). The recent application of RNA sequencing to discover extracellular RNAs in Framingham Heart Study (FHS) participants has identified over 1,000 human extracellular RNAs (ex-RNAs), including microRNAs, snoRNAs, and piRNAs, present in biofluids (Mick et al., 2017). Moreover, an independent cohort confirmed the presence of snoRNAs in the plasma. Subsequent quantitative measurements of exRNAs in participants of the FHS were undertaken to identify possible links with coronary heart disease. In 2763 FHS participants, RT-qPCR included 331 of the most abundant miRNAs, 43 snoRNAs, and 97 piRNAs, and revealed that these was no relationship between ex-RNAs and the prevalence (*n* = 286) and incidence (*n* = 69) of coronary heart disease. Nevertheless, this was the first large study of human sncRNAs beyond miRNAs to investigate the effects of ex-RNAs in relation to coronary heart disease and stroke using an unbiased approach in an observational cohort. Further investigations are required to explore the relationship between snoRNAs and coronary heart disease.

### 5.4 Role of snoRNAs in MI

MI, caused by a thrombus or coronary vascular occlusion, results in irreversible ischaemic injury in middle-aged and elderly people, but now showing a younger trend (Çakmak and Demir, 2020; Berkeley et al., 2023). MI is characterised by myocardial necrosis resulting from acute and persistent ischaemia and hypoxia of the coronary arteries (Li X et al., 2021). Additionally, MI is usually followed by cardiac remodelling, which can progress to HF in cases where the original insult is severe or prolonged (Tang et al., 2021). As a novel ncRNA, the snoRNA-mediated regulation of MI has not been systematically studied. However, Håkansson et al. reported that in a ST-elevation myocardial infarction (STEMI)–patient cohort, seven snoRNAs (SNORD112, SNORD113-2, SNORD113-6, SNORD113-7, SNORD113-8, SNORD113-9 and SNORD114-1) were detectable in plasma after hospitalisation, albeit at low levels (Hakansson et al., 2019). Furthermore, plasma SNORD113-2 was upregulated two-fold between day 4 and 30 in STEMI patients after hospitalisation. The remaining snoRNA levels showed time-dependent regulation; however, no significant changes were observed in their expression. Schena et al. demonstrated that a Cortical Bone Stem Cell-Derived Exosomes (CBSC-dEXOs) injection into the myocardial infarction region had a protective effect against ischaemia-reperfusion injury (Schena et al., 2021). They performed RNA sequencing on normal human ventricular cardiac fibroblasts (NHCFs) after human CBSC-dEXO treatment, demonstrating that cotreatment with TGFβ and exosomes decreased snoRNA levels. This may be ascribed to CBSC-derived exosomal snoRNA content reducing ribosomal stability within the treated cells, affecting protein translation and reducing fibroblast activation. However, further studies are required to confirm this hypothesis. It is well established that CVDs may lead to MI, myocardial ischaemia, and hypertrophic cardiomyopathy (HCM) (Lim et al., 2020). Therefore, in addition to evaluating the relationship between snoRNAs and MI, it may be worthwhile to determine the snoRNA-regulatory targets and signalling pathways associated with other CVDs.

### 5.5 Role of snoRNAs in HF

HF is a common CVD in the elderly, which refers to the incapacity of the heart to supply blood to the body at a rate proportional to its demands, or it can only do so at the cost of high filling pressures. The main clinical manifestations are shortness of breath, fatigue, and ankle edema (Lam et al., 2023). Welten et al. recently discovered the prominent involvement of a large gene cluster of 54 miRNAs transcribed from human chromosome 14 (14q32) in CVDs. In addition to 54 miRNAs, the human 14q32 locus also encodes 3 lncRNAs and 41 snoRNAs (Welten et al., 2016). Håkansson et al. screened the 14q32 locus within a dataset of 5,244 PROspective Study of Pravastatin in the Elderly at Risk (PROSPER) participants (Hakansson et al., 2019). Single nucleotide polymorphisms (SNPs) in the 14q32 snoRNA-cluster were associated with HF, and snoRNAs independently modulated CVD risk. Moreover, they found that, compared to naive vena saphena magna tissues and failed coronary bypasses, snoRNA expression from the DLK1-DIO3 locus on 14q32 was the highest in human end-stage HF samples. These findings demonstrated that 14q32 snoRNAs play a unique role in CVDs, and that snoRNAs merit more attention and effort in future basic and clinical cardiovascular studies.

### 5.6 Role of snoRNAs in vascular remodelling and peripheral artery disease

Vascular remodelling is a multifactorial process which includes both adaptive and maladaptive alterations of the vessel wall (Gong et al., 2021). This includes processes such as angiogenesis, arteriogenesis, atherosclerosis, hypertension, and restenosis after angioplasty. Vascular remodelling can be beneficial, such as during neovascularization after ischemia. However, vascular remodelling is also the primary underlying cause of most CVDs and is a pathological process governed by genetic and environmental interactions with a complex aetiology and pathogenesis. All layers of the arterial wall, including the tunica intima composed of endothelial cells, tunica media composed of smooth muscle cells, and tunica adventitia mainly composed of fibroblasts, play their respective roles in vascular remodelling (Seidelmann et al., 2014). However, the role of adventitial fibroblasts has often been underestimated. Van Ingen et al. demonstrated that SNORD113-6/AF357425 plays a dual role in the integrin signalling pathway and arterial fibroblast function via two distinct mechanisms: pre-mRNA processing/splicing and 2′-O-methylation of mRNA targets, which has a stabilising effect on mRNA expression (van Ingen et al., 2022b). They observed that the inhibition of SNORD113-6 altered the function of human primary arterial fibroblasts, possibly as a result of changes in the integrin signalling pathway, which is crucial for fibroblast cell–cell and cell–matrix interactions. According to their latest findings, tRNAs are the predominant small RNA targets of SNORD113-6/AF357425 in primary fibroblasts (van Ingen et al., 2022a). SNORD113-6/AF357425 induced 2′-O-methylation to protect tRNA^Leu^ against site-specific fragmentation during vascular remodelling, rather than promoting it. However, the function of tRF^Leu 47–64^ in vascular remodelling and CVDs remains unclear. Peripheral artery disease (PAD) is caused by progressive atherosclerosis and primarily affects arteries of the lower extremities (Hiatt et al., 2008; Campia et al., 2019). In patients with end-stage PAD and elite cyclists, Håkansson et al. examined the plasma levels of a specific group of snoRNAs from the 14q32 locus (Håkansson et al., 2018). They reported that SNORD114-1 were highly expressed in patients with PAD as opposed to that in elite cyclists. They demonstrated, for the first time, that endurance exercise affects the plasma levels of SNORD114-1. Continuing this line of studies, Nossent et al. showed that the 4 snoRNAs (SNORD112, SNORD113-2, SNORD113-6, SNORD114-1) were highly expressed in the plasma of 104 patients with PAD (Nossent et al., 2019). They further evaluated the relationship between snoRNAs and diseases associated with PAD prognosis, as well as classical risk factors for PAD development and progression. These findings indicated that the plasma levels of these snoRNAs were not directly related to most of the classical risk factors of atherosclerosis and restenosis in target vessels. Strong negative associations were found between the levels of SNORD113-2 and SNORD114-1 and platelet activity, which plays a key factor in the long-term outcome of PAD and CVD.

### 5.7 Role of snoRNAs in HCM

HCM is a common inheritable cardiac disorder featuring left ventricular hypertrophy (LVH) which cannot explained by other systemic, cardiac or metabolic diseases capable of leading to the degree of LVH (Desai et al., 2023). The clinical manifestation is varied with symptoms ranging from no symptoms or modest effort intolerance to severe symptoms, such as syncope or dyspnea, diastolic dysfunction, advanced HF, and sudden cardiac death. Several studies have shown that different ncRNAs play key regulatory roles in the pathophysiology of HCM (Li et al., 2019; Shahzadi et al., 2021). Accordingly, James et al. presented a transcriptomic analysis of an isogenic human-induced pluripotent stem cells (hiPSC) differentiated into cardiomyocytes (hiPSC-CMs) model of HCM (James et al., 2021). They observed differential levels of snoRNAs between wild-type (WT) and HCM hiPSC-CM EVs. Overall, 12 snoRNAs were identified, including 2 H/ACA Box snoRNAs (SNORA3B and SNORA20) and 10 C/D Box snoRNAs (SNORD6, SNOTRD116-23, SNORD116-25, SNORD116-29, SNORD18A, SNORD42A, SNORD43, SNORD58C, SNORD60, and SNORD101). Interestingly, when HCM hiPSC-CMs were exposed to increased workload, the specifically changed. When compared to 2 Hz-stimulated WT hiPSC-CM EVs, SNORD96A and SNORD73A levels in 2 Hz-stimulated HCM hiPSC-CM EVs were significantly increased. In addition, 2 Hz stimulation was sufficient to change the snoRNA levels in the HCM hiPSC-CM EVs, including SNORA12 and SNORD3A. These snoRNAs may play an important role in post-translational modifications and alternative splicing and may be used as HCM biomarkers or RNA-targeting therapies in the future. Furthermore, Tallo et al. made a *Drosophila* model of feline HCM and conducted transcriptome analyses using RNA-seq (Tallo et al., 2021). They found that snoRNAs were significantly dysregulated in exercised female flies harbouring mutant alleles as opposed to flies that harboured the WT allele. Among them, 23 snoRNAs were downregulated by *R820W*(*Arg820Trp*) while upregulated by WT just in the exercised females. These findings suggest that the identified deregulated snoRNAs may play a role in the pathogenesis of HCM, especially under cardiac stress conditions.

## 6 Future perspectives and conclusion

CVDs are the leading contributors to mortality and disability worldwide (Mensah et al., 2019). In current CVD treatment strategies, the emphasis is on disease prevention, early diagnosis, and individualised treatment. Although many CVD biomarkers, which facilitate diagnosis and monitoring, have been discovered and used, they are still unsatisfactory for the clinical diagnosis and treatment of CVDs. Therefore, identification of novel, sensitive, and effective biomarkers is becoming increasingly important. Currently, medical research is focussed on ncRNAs (Poller et al., 2018; Fasolo et al., 2019; Slack and Chinnaiyan, 2019), resulting in numerous studies demonstrating their functions and mechanisms and identifying potential CVD biomarkers (Chen et al., 2020; Smani et al., 2020; Zhu et al., 2021). However, the regulatory mechanisms connecting ncRNAs with CVDs are mainly unknown and require further research. Nevertheless, the imperative need to identify novel CVD biomarkers and targets has led to the discovery of snoRNAs, which shows significant potential in this area of research.

To date, studies on the complex regulatory mechanism of snoRNAs has generally focused on cancers and neurodegenerative diseases. However, the regulatory mechanisms of snoRNAs in CVDs, which is the focus of this review, have not been extensively investigated. Consequently, our summary is somewhat constrained. This review focuses specifically on several CVDs such as CHD, MI, PAD, and HF that may be potential regulated by snoRNAs. However, the involved snoRNAs and their underlying regulatory mechanisms in CVDs have not been fully elucidated, and further investigation is required. Through systematic reviews of snoRNA and CVDs, we found an interesting phenomenon that many snoRNAs, such as SNORD113 family and SNORD114 family, are differentially expressed in multiple CVDs including HF, MI, and PAD diseases. These interesting overlaps lead us to speculate that some snoRNA families may play important roles in different CVDs, which also indicates that snoRNA may have great potential space in the study of CVDs. At present, snoRNA can achieve promising results through RNA-seq and bioinformatics research, but there is almost rarely clear mechanism research or clinical diagnosis to support these results; Therefore, it is necessary to continue studying the role of snoRNA in CVDs. Advancements in modern molecular technology will be conducive to a comprehensive and in-depth study of the mechanism of snoRNAs in CVDs. snoRNAs are highly likely to become biomarkers and targets for CVDs diagnosis, intervention, and prognosis, thus providing new viewpoints and directions for the monitoring and treatment of CVDs. Therefore, future snoRNA research focusing on these fields deserves deeper investigation and needs increased research efforts.

### 6.1 Search strategy and selection criteria

All keywords of the searched literature: small nucleolar RNA, snoRNA, SNORD, SNORA, non-coding RNA, ncRNA, RNA, cardiovascular disease, CVD, heart, cardiac, cardiometabolic disease, CMD, congenital heart disease, CHD, congenital heart defect, coronary heart disease, Myocardial infarction, MI, heart failure, HF, vascular remodeling, peripheral artery disease, PAD, Hypertrophic cardiomyopathy, HCM, RNA-seq, RNA-seq sequencing, transcriptomics.
